# Association between diet quality scores and risk of overweight and obesity in children and adolescents

**DOI:** 10.1186/s12887-023-03966-7

**Published:** 2023-04-13

**Authors:** Xiaoyun Zheng, Hong Wang, Huiwen Wu

**Affiliations:** Department of Child Health, Maternal and Child Health Hospital of Hubei Province, No.745 Wuluo Road, Hongshan District, Wuhan, Hubei Province 430070 People’s Republic of China

**Keywords:** Diet quality, Overweight, Obesity, Children and adolescents, Association

## Abstract

**Background:**

This study examined the associations of diet quality assessed by Healthy Eating Index 2015 (HEI-2015), Alternative Healthy Eating Index 2010 (AHEI-2010), Mediterranean Diet (MedDiet) and overweight/obesity in children and adolescents.

**Methods:**

This cross-sectional study used data of participants aged 2–19 years from National Health and Nutrition Examination Survey (NHANES) 2005–2018. The weighted logistic regression model was adopted to explore the association between diet quality scores and overweight, obesity in children and adolescents. Subgroup analysis was also performed based on sex.

**Results:**

A total of 9,724 participants were included in children group (2–11 years old), and 5,934 were adolescent group (12–19 years old). All participants were divided into based on the BMI-for-age: underweight and normal, overweight and obesity groups. After adjusting for age, race, poverty-income ratio, maternal smoking during pregnancy and total energy, HEI-2015 and MedDiet scores were related to the risk of overweight in children, and only MedDiet scores remained associated with a decreased risk of obesity in children. MedDiet scores were associated with a decreased risk of overweight, obesity in adolescents, respectively, after adjusting age, sex, race, poverty-income ratio, cotinine, total energy and physical activity. The similar results in male participants were also found.

**Conclusion:**

Higher MedDiet scores were associated with lower the risk of overweight and obesity, respectively, particularly for male children and adolescents. The higher HEI-2015 scores were also related to the risk of overweight in children.

**Supplementary Information:**

The online version contains supplementary material available at 10.1186/s12887-023-03966-7.

## Background

Recently, overweight and obesity in children and adolescents has become one of the global public health challenges [1, 2]. It is estimated that the number of overweight and obesity in children and adolescents will reach 49.48 million by 2030 [3]. Obesity in childhood may contribute to the development of many chronic diseases in adulthood, including cardiovascular disease, hypertension and metabolic syndrome. Additionally, obesity in childhood might also be associated with the risk of mortality in adulthood [4]. Current treatment strategies for childhood obesity remain unsuccessful, including diet, medication, and surgery [5]. Therefore, it is important to pay attention to some modifiable factors to prevent obesity in children and adolescents.

One of the extensively studied lifestyle factors affected obesity is diet [6, 7]. In the study of Folkvord F, et al. [8], they pointed out that promoting fruit and vegetable consumption is beneficial in preventing childhood obesity; while foods high in fat, sugar and salt consumption may cause an automatic increase of activity in the brain's reward system and override homeostatic mechanisms, which increases the risk of overweight and obesity. Dietary pattern may be an alternative holistic approach compared to dietary analysis of individual foods or nutrients, and reflects the complexity of dietary intake [6]. Nowadays, several diet quality indices, including the Healthy Eating Index 2015 (HEI-2015) [9], the Alternative Healthy Eating Index 2010 (AHEI-2010) [10] and the Mediterranean Diet (MedDiet) [11], have been developed to assess overall dietary patterns. Higher diet quality may be associated with the management of obesity in adults. A cross-sectional study from three Gulf countries showed that participants with higher adherence to the MedDiet had a lower incidence of obesity [12]. In a multiethnic cohort study, compared with lower diet quality, an increase in the diet scores (HEI-2015, AHEI-2010 and MedDiet) was associated with less weight gain [13]. However, to our knowledge, there are few studies investigating the association of these diet quality scores with the risk of childhood obesity.

Here, the aim of this study was to examine the associations of diet quality and overweight/obesity in children.

## Methods

### Study participants

**Fig. 1:**
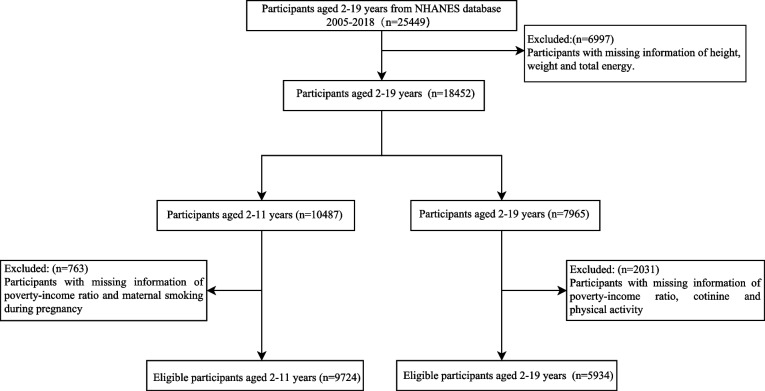
The flow diagram of the sample selection

In the cross-sectional study, we used data from the NHANES database, a large publicly available database used a complex, multi-stage probability design that represents the non-institutionalized U.S. population [14]. We included participants aged 2–19 years from NHANES database 2005–2018 in this study, and excluded some participants with missing information of height, weight and total energy. Then, a total of 18,452 participants were divided into children (2–11 years old) group and adolescent (12–19 years old) group. In the children group, we further excluded some sample with missing information of poverty-income ratio (PIR) and maternal smoking during pregnancy, resulting in a total of 9,724 participants being included. Likewise, in the adolescent group, we also excluded sample with missing information of PIR, cotinine and physical activity, 5,934 participants were included ultimately. The flow diagram of the sample selection was shown in Fig. 1. In this study, our data was accessed from NHANES (a publicly available database). For participants enrolled in NHANES, they are required to provide a written informed consent from a parent and/or legal guardian for study participation. Thus, the requirement of ethical approval for this study was waived by the Institutional Review Board of Maternal and Child Health Hospital of Hubei Province, because the data All methods were carried out in accordance with relevant guidelines and regulations (declaration of Helsinki).

### Potential confounders

Demographic data included age (years), sex, race, height (cm), weight (kg), body mass index (BMI, kg/m^2^), maternal smoking during pregnancy, PIR, total energy (kcal), cotinine (ng/mL), physical activity [Metabolic equivalent (MET)· min/day]. BMI is calculated as weight divided by height squared. Smoking status during pregnancy was determined through questionnaire for pregnant women in the NHANES: did you smoke at any time while pregnant? For participants participating in NHANES, physical activity was collected through a questionnaire. (https://wwwn.cdc.gov/Nchs/Nhanes/2007-2008/PAQ_E.htm#Appendix_1.__Suggested_MET_Scores). Participants reported the frequency and time spent doing vigorous/ moderate work-related activity, walking or bicycling for transportation, vigorous/ moderate leisure-time physical activity. MET is commonly used to describe the energy consumption while performing a specific activity [15], and physical activity was calculated as “MET × exercise time of corresponding activity/day (min/day)”.

### Assessment of diet quality

In the present study, dietary information was collected using the first 24-h dietary recall for all participants in the NHANES. The proxies or parents reported dietary intake for children aged 2–5 years and assisted in dietary interviews for children aged 6–11 years, and for adolescents aged 12–19 years, dietary intake was collected through self-report [16]. Diet quality was estimated by using three scores: HEI-2015, AHEI-2010 and MedDiet in this study.

HEI-2015 is a dietary quality index used to measure compliance of dietary intake with the Dietary Guidelines for Americans (DGA) [9]. The HEI-2015 contains thirteen dietary components, including nine adequacy components (total fruits, whole fruits, total vegetables, greens and beans, whole grains, dairy, total protein foods, seafood and plant proteins, and fatty acids) and four moderation components (refined grains, sodium, saturated fats and added sugars). A total score of all components ranges from 0 to 100, with a higher score representing higher diet quality.

AHEI-2010 is a diet quality index based on foods or food components associated with chronic disease risk [17]. In this study, AHEI-2010 is composed of nine components: fruit, vegetables, whole grains, omega-3, nuts and legumes, polyunsaturated fatty acids, sugar-sweetened beverages, red and processed meat, and sodium. The score of each component ranges from 0 to 10, with a maximum total score of 90.

MedDiet is a measure indicator of adherence to the Mediterranean dietary pattern [18]. In this study, MedDiet contains eight components: vegetables, fruits, nuts, whole grains, legumes, red and processed meats, fish and olive oil. A total score ranges from 0 to 16, with a higher score indicating higher adherence to the MedDiet. It is worth mentioning that the majority (more than 90%) of children with 2–19 years had zero alcohol intake, the calculation of the AHEI-2010 and MedDiet scores for alcohol composition was removed in the current study.

### Outcome

The outcome of this study was considered as overweight and obesity. The BMI-for-age percentile was calculated for growth charts available from the Centers for Disease Control and Prevention [19, 20]. BMI-for-age weight status categories and the corresponding percentiles: underweight (< 5th percentile), normal (≥ 5th to < 85th percentile), overweight (≥ 85th to < 95th percentile) and obesity (≥ 95th percentile).(https://www.cdc.gov/healthyweight/assessing/bmi/childrens_bmi/about_childrens_bmi.html).

### Statistical analysis

Due to a complex sampling design of the NHANES, we adopted a weighted analysis in this study: the sampling weights for interview (WTMEC2YR) and study design variables (SDMVPSU and SDMVSTRA). For the present study, we used the Mean ± standard deviation (Mean ± SD) to describe the measurement data with normal distribution, the variance test (F) to describe the comparison between groups. While The measurement data with non-normal distribution were expressed by the median and quartiles [M (Q1, Q3)], and comparison between groups used the Mann–Whitney U rank-sum test. We adopted the number cases and composition ratio n (%) to describe the enumeration data, and the comparison of groups was expressed by Chi-square test (χ^2^). Participants with missing variables were excluded from this study, and sensitivity analyses were performed on the data before and after removal of participants (Supplemental Tables 1 and 2). Python 3.10.5 was used for data cleaning and missing value processing, SAS 9.4 for statistical analysis, and R 4.2.1 software for calculation of diet scores. A *P*-value < 0.05 was considered as statistically significant.

In the current study, we conducted statistical analyses for children aged 2–11 years (*n* = 9,724) and adolescents aged 12–19 years (*n* = 5,934) separately. A weighted logistic regression model was adopted to explore the association between diet quality scores and overweight, obesity in children and adolescents. Odds ratio (OR) value with 95% confident interval (CI) were calculated. Furthermore, subgroup analysis was also performed based on sex.

## Results

### Participants’ characteristics

As shown in Table 1, 9,724 children aged 2–11 years was divided into three group based on the BMI-for-age: underweight and normal group (*n* = 6,584, 67.7%), overweight group (*n* = 1,407, 14.5%) and obesity group (*n* = 1,733, 17.8%). Of these included participants, 4,890 (50.29%) were male and 4,834 (49.71%) were female; the median age of all children was 6 (4.00, 9.00) years. There were significant differences in the distribution of age, race, PIR, maternal smoking during pregnancy, height, weight, BMI, total energy, HEI-2015 score, AHEI-2010 score, and MedDiet score. The result also indicated that age, sex, PIR, maternal smoking during pregnancy and total energy might be potential confounders which effected the relationship of diet quality scores and overweight, obesity in children.

**Table 1:** The characteristics of children aged 2–11 years

Variables	Total (*n* = 9724)	Underweight and normal group (*n* = 6584)	Overweight group (*n* = 1407)	Obesity group (*n* = 1733)	Statistics	*P*
Age, years, M (Q_1_, Q_3_)	6.00 (4.00, 9.00)	6.00 (3.00, 8.00)	7.00 (5.00, 10.00)	8.00 (6.00, 10.00)	χ^2^ = 2648.757	< 0.001
Sex, n (%)					χ^2^ = 2.711	0.258
Male	4890 (50.29)	3333 (50.62)	679 (48.26)	878 (50.66)		
Female	4834 (49.71)	3251 (49.38)	728 (51.74)	855 (49.34)		
Race, n (%)					χ^2^ = 142.034	< 0.001
Mexican American	2342 (24.08)	1431 (21.73)	393 (27.93)	518 (29.89)		
Other Hispanic	912 (9.38)	561 (8.52)	151 (10.73)	200 (11.54)		
Non-Hispanic White	3041 (31.27)	2199 (33.40)	404 (28.71)	438 (25.27)		
Non-Hispanic Black	2334 (24.00)	1552 (23.57)	336 (23.88)	446 (25.74)		
Other Race	1095 (11.26)	841 (12.77)	123 (8.74)	131 (7.56)		
PIR, M (Q_1_, Q_3_)	1.47 (0.78, 3.00)	1.55 (0.81, 3.22)	1.41 (0.78, 2.90)	1.27 (0.74, 2.37)	χ^2^ = 2648.757	< 0.001
Maternal smoking during pregnancy, n (%)					χ^2^ = 14.889	< 0.001
Yes	1220 (12.55)	799 (12.14)	157 (11.16)	264 (15.23)		
No	8504 (87.45)	5785 (87.86)	1250 (88.84)	1469 (84.77)		
Height, cm, Mean ± SD	121.31 ± 19.98	117.34 ± 19.26	126.40 ± 19.20	132.29 ± 18.17	F = 480.741	< 0.001
Weight, kg, M (Q_1_, Q_3_)	24.15 (17.50, 34.60)	20.70 (15.80, 27.90)	30.70 (21.60, 41.30)	42.70 (29.70, 55.80)	χ^2^ = 2648.757	< 0.001
BMI, kg/m^2^, Mean ± SD	17.82 ± 3.75	15.92 ± 1.35	19.20 ± 1.74	23.90 ± 4.19	F = 9441.430	< 0.001
Total energy, kcal, Mean ± SD	1738.39 ± 545.64	1700.51 ± 525.71	1805.99 ± 554.15	1827.45 ± 595.45	F = 50.254	< 0.001
HEI-2015, Mean ± SD	52.19 ± 11.45	52.61 ± 11.44	51.49 ± 11.30	51.15 ± 11.51	F = 14.207	< 0.001
AHEI-2010, Mean ± SD	30.99 ± 7.66	31.36 ± 7.63	30.58 ± 7.76	29.91 ± 7.55	F = 27.002	< 0.001
MedDiet, M (Q_1_, Q_3_)	3.00 (2.00, 4.00)	3.00 (2.00, 4.00)	3.00 (2.00, 4.00)	2.00 (1.00, 4.00)	χ^2^ = 2648.757	< 0.001

*PIR* Poverty-income ratio, *BMI* Body mass index, *HEI-2015* Healthy Eating Index 2015, *AHEI -2010* Alternative Healthy Eating Index 2010, *MedDiet* Mediterranean Diet

Similarly, we also collected the characteristics of 5,934 adolescents aged 12–19 years (Table 2). The prevalence of overweight and obesity between adolescents aged 12–19 years were 17.8% and 23.5% respectively. We found that age, sex, race, PIR, height, weight, BMI, cotinine, total energy, physical activity and MedDiet score had significant differences between three groups. Age, sex, race, PIR, cotinine, total energy and physical activity might affect the association of diet quality scores and overweight, obesity in adolescents aged 12–19 years, which were considered as confounders.

**Table 2:** The characteristics of adolescents aged 12–19 years

Variables	Total (*n* = 5934)	Underweight and normal group (*n* = 3484)	Overweight group (*n* = 1056)	Obesity group (*n* = 1394)	Statistics	*P*
Age, years, Mean ± SD	15.47 ± 2.28	15.54 ± 2.26	15.46 ± 2.31	15.33 ± 2.28	F = 4.079	0.017
Sex, n (%)					χ^2^ = 8.576	0.014
Male	3037 (51.18)	1828 (52.47)	500 (47.35)	709 (50.86)		
Female	2897 (48.82)	1656 (47.53)	556 (52.65)	685 (49.14)		
Race, n (%)					χ^2^ = 60.495	< 0.001
Mexican American	1487 (25.06)	791 (22.70)	293 (27.75)	403 (28.91)		
Other Hispanic	553 (9.32)	332 (9.53)	96 (9.09)	125 (8.97)		
Non-Hispanic White	1709 (28.80)	1076 (30.88)	294 (27.84)	339 (24.32)		
Non-Hispanic Black	1561 (26.31)	867 (24.89)	283 (26.80)	411 (29.48)		
Other Race	624 (10.52)	418 (12.00)	90 (8.52)	116 (8.32)		
PIR, M (Q_1_, Q_3_)	1.61 (0.85, 3.22)	1.73 (0.89, 3.50)	1.56 (0.85, 3.10)	1.39 (0.78, 2.75)	χ^2^ = 3174.790	< 0.001
Height, cm, Mean ± SD	165.33 ± 9.94	165.13 ± 10.17	164.93 ± 9.49	166.14 ± 9.63	F = 6.208	0.002
Weight, kg, Mean ± SD	67.14 ± 20.40	56.11 ± 10.74	70.05 ± 10.68	92.52 ± 21.04	F = 3467.562	< .001
BMI, kg/m^2^, Mean ± SD	24.37 ± 6.35	20.42 ± 2.39	25.62 ± 2.06	33.30 ± 5.84	F = 6901.474	< .001
Cotinine, ng/ml, M (Q_1_, Q_3_)	0.04 (0.01, 0.43)	0.04 (0.01, 0.35)	0.04 (0.01, 0.47)	0.06 (0.02, 0.61)	χ^2^ = 3174.790	< 0.001
Total energy, kcal, M (Q_1_, Q_3_)	1917.00 (1481.00, 2468.50)	2012.50 (1571.75, 2576.25)	1833.00 (1382.00, 2371.75)	1766.25 (1336.00, 2286.00)	χ^2^ = 3174.790	< 0.001
Physical activity, MET· min, M (Q_1_, Q_3_)	680.00 (240.00, 1300.00)	720.00 (249.75, 1320.00)	665.00 (240.00, 1410.00)	600.00 (229.50, 1200.00)	χ^2^ = 3174.790	< 0.001
HEI-2015, Mean ± SD	47.25 ± 11.32	47.30 ± 11.24	47.10 ± 11.28	47.26 ± 11.54	F = 0.129	0.879
AHEI-2010, Mean ± SD	29.17 ± 8.10	29.13 ± 8.18	29.34 ± 7.99	29.15 ± 7.99	F = 0.273	0.761
MedDiet, M (Q_1_, Q_3_)	3.00 (2.00, 4.00)	3.00 (2.00, 4.00)	3.00 (2.00, 4.00)	3.00 (2.00, 4.00)	χ^2^ = 3174.790	< 0.001

*PIR* Poverty-income ratio, *BMI* Body mass index, *MET* Metabolic equivalent, *HEI-2015* Healthy Eating Index 2015, *AHEI -2010* Alternative Healthy Eating Index 2010, *MedDiet* Mediterranean Diet

### Association of diet quality scores and overweight, obesity in children and adolescents

The relationship between diet quality assessed by HEI-2015, AHEI-2010 and MedDiet scores and overweight, obesity in children was displayed in Table 3. In unadjusted logistic regression, HEI-2015, AHEI-2010 and MedDiet scores were associated with overweight and obesity in children, respectively (Model 1). After adjusting for age, race, PIR, maternal smoking during pregnancy and total energy, HEI-2015 (OR = 0.99, 95% CI: 0.98–0.99, *P* = 0.014) and MedDiet scores (OR = 0.95, 95% CI: 0.91–0.99, *P* = 0.013) were related to the risk of overweight in children, and only MedDiet scores (OR = 0.95, 95% CI: 0.91–0.99, *P* = 0.009) remained associated with a decreased risk of obesity in children.

**Table 3:** Association of diet quality scores and overweight, obesity in children and adolescents

Population	Outcomes	Diet quality scores	Model 1		Model 2	
			**OR (95%CI)**	***P***	**OR (95%CI)**	***P***
Children aged 2–11 years	Overweight	HEI-2015	0.98 (0.98–0.99)	< 0.001	0.99 (0.98–0.99)^a^	0.014
		AHEI-2010	0.99 (0.98–0.99)	0.023	1.00 (0.99–1.01)^a^	0.876
		MedDiet	0.94 (0.90–0.98)	0.002	0.95 (0.91–0.99)^a^	0.013
	Obesity	HEI-2015	0.99 (0.98–0.99)	< 0.001	1.00 (0.99–1.00)^a^	0.548
		AHEI-2010	0.97 (0.96–0.98)	< 0.001	0.99 (0.98–1.00)^a^	0.092
		MedDiet	0.92 (0.89–0.96)	< 0.001	0.95 (0.91–0.99)^a^	0.009
Adolescents aged 12–19 years	Overweight	HEI-2015	1.00 (0.99–1.00)	0.194	0.99 (0.99–1.00)^b^	0.145
		AHEI-2010	1.00 (0.99–1.01)	0.540	0.99 (0.98–1.00)^b^	0.244
		MedDiet	0.96 (0.92–1.00)	0.069	0.96 (0.91–0.99)^b^	0.049
	Obesity	HEI-2015	0.99 (0.99–1.00)	0.187	0.99 (0.98–1.00)^b^	0.110
		AHEI-2010	1.00 (0.99–1.01)	0.472	0.99 (0.98–1.00)^b^	0.150
		MedDiet	0.97 (0.92–1.01)	0.136	0.95 (0.91–0.99)^b^	0.045

*HEI-2015* Healthy Eating Index 2015, *AHEI -2010* Alternative Healthy Eating Index 2010, *MedDiet* Mediterranean Diet, *OR* Odds ratio, *CI* Confident interval

Model 1: crude model

Model 2: ^a^adjusted age, race, maternal smoking during pregnancy and total energy

^b^adjusted age, sex, race, poverty-income ratio, cotinine, total energy and physical activity

Table 3 shows that the relationship between diet quality and overweight, obesity in adolescents. The univariate logistic analysis found that there was no significant significance between diet quality and overweight, obesity in adolescents (*P* > 0.05). The multivariate analysis indicated that MedDiet scores were associated with a decreased risk of overweight (OR = 0.96, 95% CI: 0.91–0.99, *P* = 0.049), obesity in adolescents (OR = 0.95, 95% CI: 0.91–0.99, *P* = 0.045), respectively, after adjusting age, sex, race, PIR, cotinine, total energy and physical activity.

Considering that this study focused on the risk of overweight and obesity in children and adolescents, thus the alcohol intake score in both AHEI-2010 and MedDiet were excluded. Sensitivity analysis was performed on the relationship between the AHEI-2010 and MedDiet in before and after deleting alcohol intake score and overweight and obesity in children and adolescents (Supplemental Table 3). The result showed that the diet quality scores, AHEI-2010 and MedDiet were robustly associated with the risk of overweight, obesity in children and adolescents.

### Subgroup analysis based on sex

We also assessed the associations of diet quality and overweight, obesity in children and adolescents in different sex (Table 4). For male children aged 2–11 years, HEI-2015 (OR = 0.99, 95% CI: 0.98–0.99, *P* = 0.021) and MedDiet scores (OR = 0.92, 95% CI: 0.87–0.98, *P* = 0.008) were related to the risk of overweight, and there was a correlation between MedDiet score and the risk of obesity. Among male adolescents aged 12–19 years, MedDiet scores were associated with risk of overweight (OR = 0.90, 95% CI: 0.85–0.96, *P* < 0.001), obesity (OR = 0.94, 95% CI: 0.88–0.99, *P* = 0.039), respectively. Notably, we observed that the association of diet quality scores with overweight and obesity in children and adolescents was not statistically significant (*P* > 0.05).

**Table 4:** Association of diet quality scores and overweight, obesity in children and adolescents based on sex

Population	Outcomes	Overweight				Obesity			
		**Male**		**Female**		**Male**		**Female**	
		**OR (95%CI)**	***P***	**OR (95%CI)**	***P***	**OR (95%CI)**	***P***	**OR (95%CI)**	***P***
Children aged 2–11 years^a^	HEI-2015	0.99 (0.98–0.99)	0.021	0.99 (0.99–1.00)	0.252	1.00 (0.99–1.01)	0.510	1.00 (0.99–1.01)	0.892
	AHEI-2010	1.00 (0.99–1.02)	0.826	1.00 (0.98–1.01)	0.633	0.99 (0.98–1.01)	0.310	0.99 (0.97–1.00)	0.114
	MedDiet	0.92 (0.87–0.98)	0.008	0.98 (0.93–1.03)	0.416	0.92 (0.87–0.97)	0.003	0.98 (0.92–1.04)	0.465
Adolescents aged 12–19 years^b^	AHEI-2010	0.98 (0.96–0.99)	0.008	1.01 (0.99–1.03)	0.191	1.00 (0.98–1.01)	0.716	0.99 (0.97–1.00)	0.073
	HEI-2015	0.99 (0.98–1.00)	0.233	1.00 (0.98–1.01)	0.367	0.99 (0.98–1.00)	0.235	0.99 (0.98–1.01)	0.276
	MedDiet	0.90 (0.85–0.96)	< 0.001	1.01 (0.94–1.08)	0.858	0.94 (0.88–0.99)	0.039	0.97 (0.90–1.04)	0.359

*HEI-2015* Healthy Eating Index 2015, *AHEI -2010* Alternative Healthy Eating Index 2010, *MedDiet* Mediterranean Diet, *OR* Odds ratio, *CI* Confident interval

^a^ adjusted age, race, maternal smoking during pregnancy and total energy

^b^ adjusted age, sex, race, poverty-income ratio, cotinine, total energy and physical activity

## Discussion

Using the data from the NHANES database, this study examined the associations of diet quality and overweight /obesity in children and adolescents. The result indicated that higher MedDiet scores were associated with lower the risk of overweight and obesity, respectively, with similar results in male participants. In addition, the relationship regarding higher HEI-2015 scores and the risk of overweight in children aged 2–11 years was observed.

Several previous studies have investigated diet quality scores in relation to overweight/obesity [12, 13]. MedDiet is considered as a healthy dietary pattern that combines food with antioxidants and anti-inflammatory nutrients [21]. A cross-sectional study among 481 postmenopausal women indicated an inverse relationship of MedDiet pattern and obesity indices [22]. Furthermore, in a cross-sectional study on 1,610 adolescent students aged 12–17 years, an increased adherence to MedDiet was reported to be related to a decreased waist circumference [23]. Consistent with previous research, our study suggested that adherence to MedDiet might be protective against later risk of overweight and obesity for children and adolescents. Possible explanations about the relationship of MedDiet and overweight and obesity for children are, the MedDiet is rich in dietary fiber, which may protect against weight gain by increasing satiety through prolonging chewing and releasing the cholecystokinin [24]. MedDiet rich in polyunsaturated fatty acids may inhibit inflammatory processes and enhance endothelial function [25]. It is worth mentioning that after adjusting some covariates, the relationship of MedDiet with overweight and obesity was significant among male children and male adolescents, but not for female. A population-based case–control study also showed that MedDiet was related to the risk of rheumatoid arthritis among men, but no significant association among women [26]. At present, the biological mechanisms of sex difference in the inverse association of MedDiet and overweight and obesity for male children and male adolescents is unclear. The high satiety of MedDiet contributes to the beneficial effects on the regulation of eating behavior among men, which may be related to neurobiological mechanism [27, 28]. The mechanisms of the gender difference are needed to be further considered in the future.

Likewise, this also found that the relationship of higher HEI-2015 scores and the risk of overweight in children aged 2–11 years. HEI-2015 is a diet quality index that measures the consistency of dietary guidelines for Americans [9]. Dietary quality assessed by HEI-2015 has been shown to be positively correlated with antioxidant capacity [9, 29]. According to the International Diabetes Federation (IDF) criteria, the higher the dietary antioxidant intake, the lower the likelihood of developing obesity [29]. However, this study found no significant association between the AHEI-2010 score and the risk of overweight/obesity in children, which may be related to the sample size included in this study. More research will be needed to explore the association of AHEI-2010 score and overweight/obesity in the future. To our knowledge, this study is the first to examine the association of three dietary scores with overweight and obesity by using publicly available and population-based NHANES data. This finding might provide an insight into prevention strategies for overweight/obesity in children and adolescents: greater adherence to the MedDiet may be associated with improved overweight/obesity in children and adolescents.

Nonetheless, some limitations need to be noted in interpreting the study findings. Firstly, because this was a cross-sectional study, we were unable to obtain a causal relationship between three diet scores and the risk of overweight, obesity in children and adolescents. Secondly, this study used dietary data recorded from a 24-h recall, so this dietary data may not be representative of the participants’ long-term dietary patterns. Thirdly, the Mediterranean Diet Quality Index for children and adolescents (KIDMED) has been used to assess adherence to the Mediterranean diet in children and adolescents in recent years. However, all information on participants in this study was obtained from NHANES database, we couldn't obtain the information on KIDMED. Fourth, the study lacked the information on the obesogenic environment characterized by the presence of obesity in parents and relatives, which may be a confounding factor. Lastly, even though we adjusted some covariates in this study, there may be underlying genetic factors that might affect our results. More prospective studies are needed to determine the relationship between three diet scores and the risk of overweight, obesity in children and adolescents, and elucidate the mechanisms of this association.

## Conclusion

The higher MedDiet scores were associated with lower the risk of overweight and obesity, respectively, particularly for male children and adolescents. In addition, the higher HEI-2015 scores were also related to the risk of overweight in children.

## Supplementary Information

**Additional file 1: Supplemental Table 1.** Sensitivity analyses were performed on the data before and after removal of participants in children aged 2-11 years. **Supplemental Table 2.** Sensitivity analyses were performed on the data before and after removal of participants in adolescents aged 12-19 years. **Supplemental Table 3.** Sensitivity analysis on the AHEI-2010 and MedDiet before and after deleting alcohol intake score.

## Data Availability

The datasets generated and/or analyzed during the current study are available in the NHANES database (https://www.cdc.gov/nchs/nhanes/about_nhanes.htm).

## Abbreviations

CVD: Cardiovascular disease
MedDiet: Mediterranean Diet
PIR: Poverty-income ratio
BMI: Body mass index
MET: Metabolic equivalent
DGA: Dietary Guidelines for Americans
OR: Odds ratio
CI: Confident interval
