# Supplementary material for: Association between diet quality scores and risk of overweight and obesity in children and adolescents
Source: BMC Pediatr. 2023 Apr 13;23:169. doi: 10.1186/s12887-023-03966-7 (PMC10100112; doi:10.1186/s12887-023-03966-7)
Supplement: Supplementary file 1 — Additional file 1: Supplemental Table 1. Sensitivity analyses were performed on the data before and after removal of participants in children aged 2-11 years. Supplemental Table 2. Sensitivity analyses were performed on the data before and after removal of participants in adolescents aged 12-19 years. Supplemental Table 3. Sensitivity analysis on the AHEI-2010 and MedDiet before and after deleting alcohol intake score. [file 12887_2023_3966_MOESM1_ESM.docx]

**Supplemental Table 1 Sensitivity analyses were performed on the data before and after removal of participants in children aged 2-11 years**

| **Variables** | **After removing participants (n=9724)** | **Before removing participants (n=10487)** | **Statistics** | ***P*** |
| --- | --- | --- | --- | --- |
| Age, years, n (%) |  |  | χ^2^=0.051 | 0.821 |
| ≤6 | 4954 (50.95) | 5326 (50.79) |  |  |
| >6 | 4770 (49.05) | 5161 (49.21) |  |  |
| Sex, n (%) |  |  | χ^2^=0.030 | 0.863 |
| Male | 4890 (50.29) | 5261 (50.17) |  |  |
| Female | 4834 (49.71) | 5226 (49.83) |  |  |
| Race, n (%) |  |  | χ^2^=4.162 | 0.385 |
| Mexican American | 2342 (24.08) | 2572 (24.53) |  |  |
| Other Hispanic | 912 (9.38) | 1026 (9.78) |  |  |
| Non-Hispanic White | 3041 (31.27) | 3148 (30.02) |  |  |
| Non-Hispanic Black | 2334 (24.00) | 2536 (24.18) |  |  |
| Other Race | 1095 (11.26) | 1205 (11.49) |  |  |
| PIR, M (Q_1_, Q_3_) | 1.47 (0.78, 3.00) | 1.47 (0.78, 3.01) | Z=-0.211 | 0.833 |
| Maternal smoking during pregnancy, n (%) |  |  | χ^2^=0.350 | 0.554 |
| Yes | 1220 (12.55) | 1273 (12.27) |  |  |
| No | 8504 (87.45) | 9101 (87.73) |  |  |
| Height, cm, Mean ± SD | 121.31 ± 19.98 | 121.31 ± 19.98 | t=0.02 | 0.983 |
| Weight, kg, M (Q_1_, Q_3_) | 24.15 (17.50, 34.60) | 24.20 (17.40, 34.70) | Z=-0.127 | 0.899 |
| BMI, kg/m^2^, Mean ± SD | 17.82 ± 3.75 | 17.84 ± 3.76 | t=-0.31 | 0.758 |
| Total energy, kcal, Mean ± SD | 1738.39 ± 545.64 | 1734.07 ± 545.10 | t=0.56 | 0.573 |
| HEI-2015, Mean ± SD | 52.19 ± 11.45 | 52.26 ± 11.51 | t=-0.48 | 0.631 |
| AHEI-2010, Mean ± SD | 30.99 ± 7.66 | 30.99 ± 7.65 | t=0.00 | 0.997 |
| MedDiet, M (Q1, Q3) | 3.00 (2.00, 4.00) | 3.00 (2.00, 4.00) | Z=-0.737 | 0.461 |

Note: PIR, poverty-income ratio; BMI, body mass index; HEI-2015, Healthy Eating Index 2015; AHEI -2010, Alternative Healthy Eating Index 2010; MedDiet, Mediterranean Diet.

**Supplemental Table 2 Sensitivity analyses were performed on the data before and after removal of participants in adolescents aged 12-19 years**

| **Variables** | **After removing participants (n=5934)** | **Before removing participants (n=7965)** | **Statistics** | ***P*** |
| --- | --- | --- | --- | --- |
| Age, years, n (%) |  |  | χ^2^=1.497 | 0.221 |
| ≤15 | 2999 (50.54) | 4109 (51.59) |  |  |
| >15 | 2935 (49.46) | 3856 (48.41) |  |  |
| Sex, n (%) |  |  | χ^2^=1.035 | 0.309 |
| Male | 3037 (51.18) | 4007 (50.31) |  |  |
| Female | 2897 (48.82) | 3958 (49.69) |  |  |
| Race, n (%) |  |  | χ^2^=6.369 | 0.173 |
| Mexican American | 1487 (25.06) | 1964 (24.66) |  |  |
| Other Hispanic | 553 (9.32) | 710 (8.91) |  |  |
| Non-Hispanic White | 1709 (28.80) | 2210 (27.75) |  |  |
| Non-Hispanic Black | 1561 (26.31) | 2160 (27.12) |  |  |
| Other Race | 624 (10.52) | 921 (11.56) |  |  |
| PIR, M (Q1, Q3) | 1.61 (0.85, 3.22) | 1.63 (0.87, 3.24) | Z=-0.581 | 0.561 |
| Height, cm, Mean ± SD | 165.33 ± 9.94 | 165.00 ± 10.01 | t=1.91 | 0.056 |
| Weight, kg, Mean ± SD | 67.14 ± 20.40 | 66.73 ± 20.52 | t=1.18 | 0.236 |
| BMI, kg/m^2^, Mean ± SD | 24.37 ± 6.35 | 24.31 ± 6.40 | t=0.57 | 0.566 |
| Cotinine, ng/ml, M (Q_1_, Q_3_) | 0.04 (0.01, 0.43) | 0.04 (0.01, 0.40) | Z=0.653 | 0.514 |
| Total energy, kcal, M (Q_1_, Q_3_) | 1917.00 (1481.00, 2468.50) | 1904.00 (1469.50, 2460.00) | Z=1.116 | 0.264 |
| Physical activity, MET· min, M (Q_1_, Q_3_) | 680.00 (240.00, 1300.00) | 648.00 (240.00, 1280.00) | Z=1.308 | 0.191 |
| HEI-2015, Mean ± SD | 47.25 ± 11.32 | 47.17 ± 11.40 | t=0.42 | 0.675 |
| AHEI-2010, Mean ± SD | 29.17 ± 8.10 | 29.43 ± 8.13 | t=-1.86 | 0.062 |
| MedDiet, M (Q1, Q3) | 3.00 (2.00, 4.00) | 3.00 (2.00, 4.00) | Z=-1.173 | 0.241 |

Note: PIR, poverty-income ratio; BMI, body mass index; MET, metabolic equivalent; HEI-2015, Healthy Eating Index 2015; AHEI -2010, Alternative Healthy Eating Index 2010; MedDiet, Mediterranean Diet.

**Supplemental Table 3 Sensitivity analysis on the AHEI-2010 and MedDiet before and after deleting alcohol intake score**

|  | **Outcomes** | **Diet quality scores** | **Children aged 2-11 years** | | **Adolescents aged 12-19 years** | |
| --- | --- | --- | --- | --- | --- | --- |
|  |  |  | **OR (95%CI)** | ***P*** | **OR (95%CI)** | ***P*** |
| After deleting alcohol intake score | Overweight | AHEI-2010 | 1.00 (0.99-1.01) ^#^ | 0.876 | 0.99 (0.98-1.00) ^*^ | 0.244 |
|  |  | Med scores | 0.95 (0.91-0.99) ^#^ | 0.013 | 0.96 (0.91-0.99) ^*^ | 0.050 |
|  | Obesity | AHEI-2010 | 0.99 (0.98-1.00) ^#^ | 0.092 | 0.99 (0.98-1.00) ^*^ | 0.150 |
|  |  | Med scores | 0.95 (0.91-0.99) ^#^ | 0.009 | 0.95 (0.91-0.99) ^*^ | 0.045 |
| Before deleting alcohol intake score | Overweight | AHEI-2010 | 1.00 (0.99-1.01) ^#^ | 0.888 | 0.99 (0.98-1.00) ^*^ | 0.226 |
|  |  | Med scores | 0.95 (0.91-0.99) ^#^ | 0.013 | 0.96 (0.92-1.00) ^*^ | 0.050 |
|  | Obesity | AHEI-2010 | 0.99 (0.98-1.00) ^#^ | 0.093 | 0.99 (0.98-1.00) ^*^ | 0.096 |
|  |  | Med scores | 0.95 (0.91-0.99) ^#^ | 0.009 | 0.95 (0.91-0.99) ^*^ | 0.038 |

Note: HEI-2015, Healthy Eating Index 2015; AHEI -2010, Alternative Healthy Eating Index 2010; MedDiet, Mediterranean Diet; OR, odds ratio; CI, confident interval;

^#^ adjusted age, race, maternal smoking during pregnancy and total energy;

* adjusted age, sex, race, poverty-income ratio, cotinine, total energy and physical activity.
